# Thiol‐Functionalized Conjugated Metal–Organic Frameworks for Stable and Efficient Perovskite Photovoltaics

**DOI:** 10.1002/advs.202305572

**Published:** 2023-11-09

**Authors:** Xiao Liang, Mriganka Singh, Fei Wang, Patrick W. K Fong, Zhiwei Ren, Xianfang Zhou, Xuejuan Wan, Carolin M. Sutter‐Fella, Yumeng Shi, Haoran Lin, Quanyao Zhu, Gang Li, Hanlin Hu

**Affiliations:** ^1^ State Key Laboratory of Advanced Technology for Materials Synthesis and Processing, School of Materials Science and Engineering Wuhan University of Technology Wuhan 430070 China; ^2^ Hoffmann Institute of Advanced Materials Shenzhen Polytechnic 7098 Liuxian Boulevard Shenzhen 518055 China; ^3^ Molecular Foundry Division Lawrence Berkeley National Laboratory Berkeley CA 94720 USA; ^4^ The Hong Kong Polytechnic University Shenzhen Research Institute Guangdong Shenzhen 518057 China; ^5^ Department of Electronic and Information Engineering, Research Institute for Smart Energy (RISE) The Hong Kong Polytechnic University Hung Hom Kowloon Hong Kong 999077 China; ^6^ Shenzhen Key Laboratory of Polymer Science and Technology, College of Materials Science and Engineering Shenzhen University Shenzhen 518060 China; ^7^ International Collaborative Laboratory of 2D Materials for Optoelectronics Science and Technology of Ministry of Education, Institute of Microscale Optoelectronics Shenzhen University Shenzhen 518060 China

**Keywords:** in situ GIWAXS, MOF/perovskite structure, perovskite solar cells, thiol‐functionalized MOF

## Abstract

Metal–organic frameworks (MOFs) have been investigated recently in perovskite photovoltaics owing to their potential to boost optoelectronic performance and device stability. However, the impact of variations in the MOF side chain on perovskite characteristics and the mechanism of MOF/perovskite film formation remains unclear. In this study, three nanoscale thiol‐functionalized UiO‐66‐type Zr‐based MOFs (UiO‐66‐(SH)_2_, UiO‐66‐MSA, and UiO‐66‐DMSA) are systematically employed and examined in perovskite solar cells (PSCs). Among these MOFs, UiO‐66‐(SH)_2_, with its rigid organic ligands, exhibited a strong interaction with perovskite materials with more efficient suppression of perovskite vacancy defects. More importantly, A detailed and in‐depth discussion is provided on the formation mechanism of UiO‐66‐(SH)_2_‐assisted perovskite film upon in situ GIWAXS performed during the annealing process. The incorporation of UiO‐66‐(SH)_2_ additives substantially facilitates the conversion of PbI_2_ into the perovskite phase, prolongs the duration of stage I, and induces a delayed phase transformation pathway. Consequently, the UiO‐66‐(SH)_2_‐assisted device demonstrates reduced defect density and superior optoelectronic properties with optimized power conversion efficiency of 24.09% and enhanced long‐term stability under ambient environment and continuous light illumination conditions. This study acts as a helpful design guide for desired MOF/perovskite structures, enabling further advancements in MOF/perovskite optoelectronic devices.

## Graphical abstract

The study systematically employed and examined three nanoscale thiol‐functionalized UiO‐66‐type Zr‐based MOFs (UiO‐66‐(SH)_2_, UiO‐66‐MSA, and UiO‐66‐DMSA) in perovskite solar cells and provided a detailed and in‐depth discussion on the formation mechanism of UiO‐66‐(SH)_2_‐assisted perovskite film upon in situ GIWAXS performed during the annealing process. The final device improved opto‐electronic properties by over 24%.

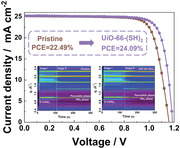

## 1 Introduction

Organic‐inorganic hybrid perovskite solar cells (PSCs) have attracted widespread interest owing to their exceptional power conversion efficiency (PCE), cost‐effectiveness, and simple processability, garnering significant attention from the scientific community.^[^ ^1^, ^2^, ^3^, ^4^, ^5^, ^6^, ^7^ ^]^ Metal‐organic frameworks (MOFs) are host matrices constructed through the assembly of metal clusters and organic ligands, showcasing a wide range of chemistries, functional capabilities, topological configurations, and robustness.^[^ ^8^, ^9^, ^10^ ^]^ Since its debut publication in 2014,^[^ ^11^ ^]^ various studies on MOF‐ PSCs have been reported, showing significantly improved device stability and optoelectronic performance.^[^ ^12^, ^13^, ^14^, ^15^, ^16^ ^]^ Wu and co‐workers^[^ ^17^ ^]^ have designed a new thiol‐functionalized 2D conjugated MOF as an electron‐extraction layer at the perovskite/cathode interface, which promotes long‐term stability and reduces the potential of lead (Pb^2+^) ion leakage. The fabricated devices exhibit a high PCE of 22.02% and retain over 90% of their original efficiency under continuous illumination maximum power point tracking for 1000 h at 85 °C. Dou et al.^[^ ^18^ ^]^ have inserted Eu‐MOF into the interface layer of PSCs, which suppresses defects, improves carrier transport, enhances light usage, and reduces ultraviolet radiation‐induced degradation. The optimized devices attain a PCE of 22.16% and exhibit excellent stability. Dong and colleagues^[^ ^19^ ^]^ have proposed a unique chemical doping approach using a series of polyoxometalates@MOFs (POM@MOF) host‐guest nanostructured dopants that quantitatively and precisely oxidize Spiro‐OMeTAD in an inert environment. The optimized POM@MOF‐doped device with a PCE of 21.5% is achieved, and the device's stability under humid conditions is markedly improved.

More importantly, functionalizing the organic ligands component of MOFs with various functional groups offers the possibility of flaw passivation for perovskite materials. Furthermore, the extremely crystalline and diverse active sites of MOF materials facilitate perovskite crystallization. Qiu et al.^[^ ^20^ ^]^ developed formic acid‐functionalized 2D MOFs as the terminating agent to passivate perovskite film grain. 2D MOFs firmly cover the surface of PbI_2_‐terminated perovskite grains, stabilizing the perovskite phases and promoting grain adhesion through strong interactions between exposed active sites and PbI_2_. The MOF‐assisted perovskite films exhibit a uniform morphology, decreased defect density, enhanced optoelectronic characteristics, and improved long‐term stability. Wang and colleagues^[^ ^21^ ^]^ selected the small molecule additive with a terminal rich in uncoordinated N groups as ligands and Zn ions as metal clusters in order to self‐assemble a 1D MOF structure. The MOF inherited functional groups from ligand molecules and was arrayed along a definite orientation, which may efficiently passivate unsaturated Pb ions of perovskite. The long‐range ordered structure can also dramatically improve the crystallization and durability of perovskite films. Consequently, the unencapsulated MOF‐modified device attained a remarkable PCE of 23.14%, and the thermal stability and continuous light illumination stability were significantly increased compared to ligand‐modified devices. Dong and co‐workers^[^ ^22^ ^]^ incorporated a double ligand into the fabrication of a novel 3D MOF material for perovskite devices, which not only has more passivation sites in the framework but also has superior chemical and thermal stability. The device boosted PCE to 22.18%. Among all the MOF compounds with multiple metal clusters, Zr‐based MOFs typically exhibit remarkable chemical, thermal, and water/moisture stability owing to their robust Zr–O bonds and multinuclear secondary building units (SBUs).^[^ ^23^ ^]^ However, few MOF‐assisted studies have evaluated the side‐chain differences between rigid conjugated benzene rings and flexible alkyl chains, and the formation mechanism of perovskite film with MOF‐assisted methods remains unclear.

Herein, we conducted a comprehensive evaluation of the impact of three thiol‐functionalized UiO‐66‐type nanoscales (≈100 nm) Zr‐based MOFs (UiO‐66‐(SH)_2_, UiO‐66‐MSA, and UiO‐66‐DMSA) added to the PbI_2_ precursor in a 2‐step perovskite fabrication process on perovskite photovoltaics. The Supporting Information provides detailed information on the morphology, size distribution, X‐ray diffraction patterns (XRD), and N_2_ absorption‐desorption isotherms of three Zr‐based MOF materials (Figure S1, Supporting Information). The X‐ray photoelectron spectroscopy (XPS) and density functional theory (DFT) calculations provide experimental and theoretical support, demonstrating that conjugated UiO‐66‐(SH)_2_‐assisted perovskite films exhibit strong interactions to form the Pb‐S bond and the I‐H bond, indicating more efficient passivation of perovskite surface defects and protection of perovskite films against degradation. For the first time, we discuss in‐depth the formation mechanism of UiO‐66‐(SH)_2_‐assisted perovskite film via in situ grazing‐incidence wide‐angle scattering measurements (GIWAXS) performed during the annealing process. MOF‐assisted perovskite films not only achieve complete conversion of PbI_2_ to perovskite phase, but also delay the crystallization kinetics of perovskite films to enhance grain expansion, which may be attributed to the modulated molecular interactions between UiO‐66‐(SH)_2_ additives and solution precursors. Ultimately, the UiO‐66‐(SH)_2_‐assisted device exhibited a PCE of 24.09%. The unencapsulated devices exhibit enhanced long‐term stability under both the ambient environment and continuous light illumination conditions. The elucidation of the MOF‐assisted perovskite film formation mechanism and the relative phase transformation process in this study will guide the desired MOF/perovskite structure.

## 2 Results and Discussion

**Figure 1:**
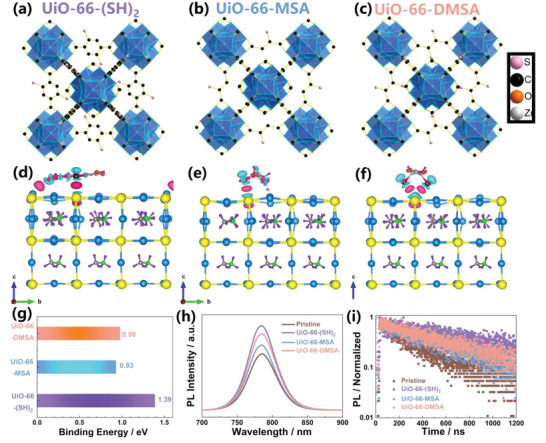
The crystal structures schematic of a) UiO‐66‐(SH)_2_, b) UiO‐66‐MSA, and c) UiO‐66‐DMSA. H atoms have been hidden for clarity. Optimized crystal structures and charge accumulation (pink) and depletion (cyan) of MAPbI_3_ (001) surface with side‐chain of d) UiO‐66‐(SH)_2_, e) UiO‐66‐MSA, and f) UiO‐66‐DMSA. g) The calculated binding energies result. h) PL and i) TRPL spectra of the pristine, UiO‐66‐(SH)_2_, UiO‐66‐MSA, and UiO‐66‐DMSA assisted perovskite films.

The three Zr‐based MOFs (UiO‐66‐(SH)_2_, UiO‐66‐MSA, and UiO‐66‐DMSA) employ conventionally available small molecule thiol‐functionalized organic ligands, namely 2,5‐dimercapto‐1,4‐benzenedicarboxylic acid (SH), mercaptosuccinic acid (MSA), and meso‐dimercaptosuccinic acid (DMSA), respectively (**Figure** **1**a–c; Figure S2a–c, Supporting Information). The SH organic ligands exhibit conjugation‐induced rigidity, with the benzene and acid parts of the SH molecule on the same platform, whereas the MSA and DMSA molecules exhibit a three‐dimensional conformation due to the flexibility of the alkyl chain, as evidenced by the optimal structure with electrostatic surface potential (ESP) (Figure S2d–f, Supporting Information). Furthermore, in our previous work, we have already investigated the capability of UiO‐66 to effectively suppress the formation of iodine vacancies on the perovskite surface via DFT calculation.^[^ ^24^ ^]^ The interaction of side chains with the perovskite (001) surface was estimated in order to simplify the DFT calculations. The detailed DFT information, including models, functionals, and other computational parameters, is shown in the supporting information. Optimized crystal structures with charge accumulation (pink) and depletion (cyan) were depicted in Figure 1d–f. While three thiol‐functionalized side‐chain molecules could interact with the Pb^2+^ ions on the perovskite surface, an I‐H bond was formed through the interaction between the H^+^ atom on the side‐chain of UiO‐66‐(SH)_2_ and the I^−^ atom on the perovskite surface. The binding energies of perovskite surface interaction with UiO‐66‐(SH)_2_ were determined to be 1.39 eV, which is higher than the values of 0.93 eV with UiO‐66‐MSA and 0.98 eV with UiO‐66‐DMSA (Figure 1g). In addition, steady‐state photoluminescence (PL) and time‐resolved photoluminescence (TRPL) were utilized to evaluate the charge transfer and recombination kinetics at the MOF/perovskite interface. The UiO‐66‐(SH)_2_ assisted perovskite film exhibited stronger PL emission (Figure 1h) and a longer TRPL charge carrier lifetime (Figure 1i; Figure S3, Supporting Information) than pristine, UiO‐66‐MSA and UiO‐66‐DMSA assisted perovskite films. This suggests that UiO‐66‐(SH)_2_ can passivate Pb^2+^ and I^−^ defects on the perovskite surface more efficiently, reduce nonradiative recombination at the UiO‐66‐(SH)_2_/perovskite interface, and enhance perovskite film quality.

**Figure 2:**
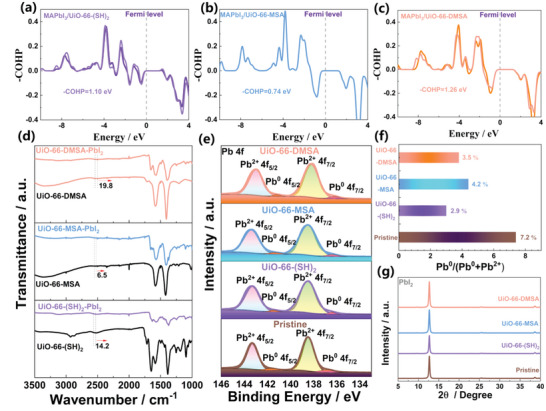
a–c) The COHP illustrated the stability of Pb‐S bonds for the adsorption of a) UiO‐66‐(SH)_2_, b) UiO‐66‐MSA, and c) UiO‐66‐DMSA on the perovskite surface, where negative values (‐COHP) represent antibonding interactions (downside), positive values represent bonding interactions (upside), and zero represents the Fermi level. d) FTIR spectra of the films from equimolar ratios of PbI_2_ and UiO‐66‐(SH)_2_, UiO‐66‐MSA, and UiO‐66‐DMSA, respectively. e) XPS measurements of Pb 4f, f) The fitted results of the Pb^0^/(Pb^0^+Pb^2+^) ratio, and g) X‐ray diffraction spectra (XRD) of PbI_2_ film.

The Crystal Orbital Hamilton Population (COHP) analysis has been developed as a reliable and effective tool for obtaining chemical‐bonding information from various types of electronic structure calculations in quantum chemistry.^[^ ^25^ ^]^ COHP is generally used to represent bonding and antibonding interactions within a system. Higher antibonding states at the Fermi level indicate lower chemical stability of the system. In this study, we utilized COHP to investigate the durability of the Pb‐S bond between the thiol group of three UiO‐66‐type MOFs and Pb ions on the perovskite surface (**Figure** **2**a–c). Our findings show that the ‐COHP for the Pb‐S bonds at the interface between perovskite and UiO‐66‐DMSA turns out to be 1.26 eV(mainly contributed by the bonding states ranging from −5.0 to −2.5 eV), which is higher than the value of 1.10 and 0.74 eV for the surface of UiO‐66‐(SH)_2_ and UiO‐66‐MSA, respectively. This result suggests that the incorporation of UiO‐66‐DMSA has improved the Pb‐S bond stability of perovskite materials. Fourier transform infrared (FTIR) spectra (as shown in Figure 2d) were used to verify the COHP calculation. The *S*–*H* stretching vibration peaks (≈2550 cm^−1^) were found to be shifted for UiO‐66‐DMSA‐PbI_2_ (19.8 cm^−1^) compared to of UiO‐66‐(SH)_2_‐PbI_2_ (14.2 cm^−1^) and UiO‐66‐MSA‐PbI_2_ (6.5 cm^−1^). Furthermore, X‐ray photoelectron spectroscopy (XPS) characterization was used to further investigate the chemical impact of the MOF on the perovskite surface. The entire spectra and I 3d spectra are shown in Figure S4a,b (Supporting Information), and the surface Pb 4f core level of the corresponding pristine samples is presented in Figure 2e. The dominating peaks (138.3 eV for 4f_7/2_ and 143.2 eV for 4f_5/2_) are associated with the Pb^2+^ components with saturated coordination, and the small peaks at lower binding energy (136.8 eV for 4f_7/2_ and 141.7 eV for 4f_5/2_) are attributed to metallic Pb cluster (Pb^0^).^[^ ^26^ ^]^ Compared with the pristine sample, the PbI_2_ films that were assisted by three UIO‐66‐type MOFs all exhibited a lower energy level shift of ≈0.2, ≈0.1, and ≈0.4 eV, respectively, corresponding to UiO‐66‐(SH)_2_, UiO‐66‐MSA, and UiO‐66‐DMSA. The results of our study suggest that the UiO‐66‐DMSA‐assisted PbI_2_ film exhibits the most significant red shift of the S‐H stretching vibration and the highest shift of the Pb 4f peak toward low binding energy, indicating the strongest coordination of the Pb─S bond. This observation is consistent with COHP results. Moreover, we observed a shift of all I 3d peaks to a lower energy position (Figure S4b, Supporting Information) in the UiO‐66‐(SH)_2_ assisted PbI_2_ film, indicating the formation of H‐I bonds. In contrast, no peak shift was observed for the other samples, which is in agreement with the calculated binding energy results (Figure 1g). The presence of three UIO‐66‐type MOFs in the target film was verified by the characteristic peaks for S^2−^ at ≈165.3 eV (Figure S4c, Supporting Information).^[^ ^27^ ^]^ We also evaluated the ratio of Pb^0^/(Pb^0^+Pb^2+^) for both pristine (7.2%), UiO‐66‐(SH)_2_ assisted (2.9%), UiO‐66‐MSA assisted (4.2%), and UiO‐66‐DMSA assisted (3.5%) samples based on the corresponding integrated peak areas from the XPS spectra (Figure 2f). This result indicates that metallic Pb^0^ was successfully suppressed via MOF‐assisted, and UiO‐66‐(SH)_2_‐assisted suppression is most effective. The UV–vis absorption spectra of the pristine and MOF‐assisted PbI_2_ and perovskite thin films showed no noticeable change (Figure S5, Supporting Information). Crystal structures and orientation of pristine and MOF‐assisted PbI_2_ thin films were evaluated by XRD (Figure 2g) and ex situ GIWAXS (Figure S6, Supporting Information). Both PbI_2_ films show a strong diffraction peak at 12.8° and the scattering peak located at *q* = 0.91 Å^−1^, corresponding to the (001) lattice plane of crystallized PbI_2_.^[^ ^28^ ^]^ This result shows the addition of MOF has not changed the crystal structure or orientation of PbI_2_ crystals.

**Figure 3:**
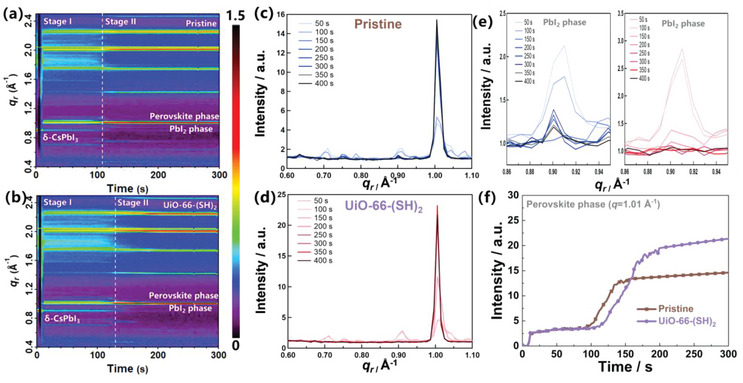
Time evolution of operando synchrotron radiation‐based GIWAXS data plotted as azimuthally integrated line profiles and corresponding GIWAXS intensity profiles along the *q*_r_ direction measured points every 50 s for the a,c) pristine and b,d) UiO‐66‐(SH)_2_‐assisted perovskite film. e) A magnified view of c,d) at *q*  = 0.91 Å^–1^ (PbI_2_ phase) for pristine (left) and UiO‐66‐(SH)_2_‐assisted perovskite film (right). f) The extracted intensity as a function of time for *q*  = 1.01 Å^−1^ (perovskite phase).

A time‐resolved GIWAXS experiment was conducted to gain insight into the effect of UiO‐66‐(SH)_2_ on the formation of perovskite thin films during the annealing process. The measurements were performed on a custom‐built spin coater operated at the Advanced Light Source, Lawrence Berkeley National Laboratory,^[^ ^29^ ^]^ and the experimental details are provided in the Supporting Information. **Figure** **3**a–d depicts the temporal evolution of the GIWAXS signal and the intensity profiles along the *q_r_* direction for both pristine and UiO‐66‐(SH)_2_‐assisted perovskite films. Stages I and II cover the crystallization process of perovskite films during the annealing process after spin coating the organic salt solution. Stage I is characterized by the presence of PbI_2_, δ‐CsPbI_3_,^[^ ^30^ ^]^ and DMF/DMSO intermediate phase,^[^ ^31^, ^32^, ^33^ ^]^ and stage II involves the phase transformation from the intermediate phase to perovskite phase and the formation of the monohydrate phase at q  = 0.61 Å.^−1[^ ^34^, ^35^ ^]^ For the pristine sample (Figure 3a,c,f), stage I consists of the PbI_2_, δ‐CsPbI_3,_ and DMF/DMSO intermediate phase with a low scattering intensity. It takes ≈144 s to be converted into the perovskite phase, accompanied by the gradual disappearance of the δ‐CsPbI_3_ (*q*  =  0.71 Å^−1^) and DMF intermediate phase (q  =  0.75 Å^−1^).^[^ ^31^ ^]^ In contrast, the UiO‐66‐(SH)_2_‐assisted perovskite film undergoes a relatively slower conversion process, with stage II appearing at ≈178 s, indicating a delay in conversion kinetics in the presence of UiO‐66‐(SH)_2_. This could be attributed to the interaction between SH^−^ within UiO‐66‐(SH)_2_ and Pb^2+^, exerting an influence on the perovskite crystal growth. In the early stages of crystal growth within the solution, SH^−^ coordinates with Pb^2+^. Subsequently, through the annealing process, SH^−^‐ Pb^2+^ is progressively replaced by Pb^2+^‐ I^−^, thereby decelerating the crystalline kinetics of the perovskite film. Ultimately, SH^−^‐ Pb^2+^ is entirely substituted by Pb^2+^‐ I^−^, culminating in the completion of perovskite crystal growth.^[^ ^32^, ^36^ ^]^ Furthermore, we captured photographic records throughout the process for both the pristine and UiO‐66‐(SH)_2_‐assisted samples, as presented in Figure S7 (Supporting Information). In comparison to the pristine, the UiO‐66‐(SH)_2_‐assisted samples exhibited a slower time duration before undergoing darkening, which is consistent with the in situ GIWAXS absorption spectroscopic results reported earlier. Importantly, the UiO‐66‐(SH)_2_‐assisted film exhibits a slower transformation and an almost negligible scattering peak of the PbI_2_ phase (Figure 3e), indicating a rapid conversion and minimal PbI_2_ residues in the final perovskite film (Figures s8 and S9, Supporting Information). These findings are consistent with our previously reported results.^[^ ^24^ ^]^

**Figure 4:**
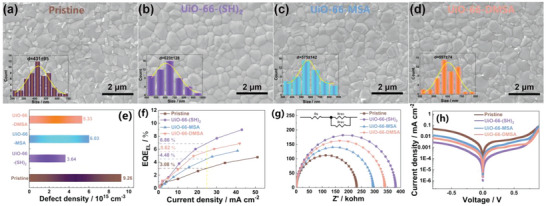
a) The plan‐view SEM and grain size distribution histograms of the a) pristine, b) UiO‐66‐(SH)_2_, c) UiO‐66‐MSA, and d) UiO‐66‐DMSA assisted perovskite thin films. e) Trap density (N_t_), f) EQE_EL_ versus current density, g) Nyquist plot of EIS, and h) The dark *J*–*V* curves of the PSCs based on pristine, UiO‐66‐(SH)_2_, UiO‐66‐MSA, and UiO‐66‐DMSA.

By analyzing the morphology of perovskite films, we were able to confirm the impact of MOF crystals on perovskite thin film morphology. Plan‐view scanning electron micrographs (SEM) of the pristine and three UiO‐66‐types MOF‐assisted perovskite thin films are shown in **Figure** **4**a–d. The SEM revealed that the apparent average grain size from SEM of pristine, UiO‐66‐(SH)_2_, UiO‐66‐MSA, and UiO‐66‐DMSA assisted perovskite films were ≈431, 623, 575, and 597 nm, respectively. Additionally, the plan‐view morphology of the corresponding PbI_2_ thin films was characterized by SEM, as shown in Figure S10a–d (Supporting Information). It is evident that the addition of MOF has transformed the morphology of PbI_2_ thin films into more pin‐holes (Figure S9b–d, Supporting Information) to establish better contact with organic salt during second‐step deposition, which is in good agreement with a previous study.^[^ ^24^ ^]^ Additionally, atomic force microscope (AFM) images confirm the evolution of the PbI_2_ film morphology, as evidenced by the heightened surface roughness, which signifies a rougher and porous film (Figure S11, Supporting Information). To quantify the level of trap states, we employed the space‐charge‐limited current (SCLC) model in hole‐only device configurations (Figure S12, Supporting Information). The defect density (N_t_) value of the UiO‐66‐(SH)_2_‐assisted device (3.64 × 10^15^ cm^–3^) is lower than that of the pristine (9.26 × 10^15^ cm^−3^), UiO‐66‐MSA‐assisted(6.03 × 10^15^ cm^−3^), and UiO‐66‐DMSA‐assisted device (5.33 × 10^15^ cm^−3^) (Figure 4e), indicating effective suppression of trap states by UiO‐66‐(SH)_2_‐assisted. As shown in Figure 4f, the UiO‐66‐(SH)_2_‐assisted PSCs showed significantly higher EQE_EL_ (6.68% at a current density of 25 mA·cm^−2^) and EL intensities at different bias voltages (Figure S13, Supporting Information) compared to the pristine (3.08%), UiO‐66‐MSA‐assisted (4.48%), and UiO‐66‐DMSA‐assisted sample (5.62%). This result suggests that the UiO‐66‐(SH)_2_‐assisted device shows inhibited nonradiative recombination. To gain insight into the carrier dynamics of the devices, electrochemical impedance spectroscopy (EIS) was performed in the dark at an applied bias of 0 V. The Nyquist plots of the PSCs in Figure 4g show that the UiO‐66‐(SH)_2_‐assisted sample has a higher charge recombination resistance (*R*_rec_) than the pristine, UiO‐66‐MSA, and UiO‐66‐DMSA. The larger *R*_rec_ values can be attributed to the lower defect density and minor carrier recombination in the respective perovskite films. Moreover, the dark current density of the PSC based on UiO‐66‐(SH)_2_‐assisted perovskite film is nearly one order of magnitude lower than the other three samples, as shown in Figure 4h, indicating a remarkable reduction in charge recombination and leakage current in the cell. The low leakage current contributes to a higher fill factor (FF).^[^ ^37^ ^]^

**Figure 5:**
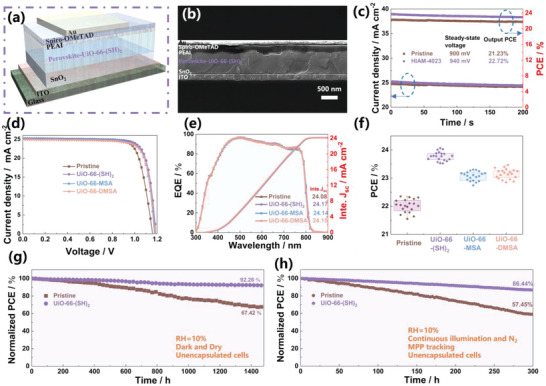
a) Schematic of PSCs and b) cross‐sectional SEM micrographs with a structure of ITO/SnO_2_/ UiO‐66‐(SH)_2_‐assisted perovskite/Spiro‐OMeTAD/Au. c) The stabilized power outputs of the pristine and UiO‐66‐(SH)_2_‐assisted devices were measured at voltages of 0.90 and 0.94 V in the ambient environment, respectively. d) Current density–voltage (*J–V*) curves under an AM1.5G solar simulator for devices. e) Corresponding EQE spectra. f) Statistic diagrams of PCE from 20 individual devices for pristine and three MOF assisted. g) Normalized PCEs of non‐encapsulated devices exposed to dry conditions (10% relative humidity) in the dark. h) Stability tests of unencapsulated solar cell devices exposed to continuous illumination (1 sun) near the (MPP) with a white LED lamp at 20.8 °C in a nitrogen atmosphere.

To verify the photovoltaic performance of the device, planar PSCs were fabricated with an ITO/ SnO_2_/ UiO‐66‐(SH)_2_‐assisted perovskite/ PEAI/ Spiro‐OMeTAD/ Au configuration (**Figure** **5**a). The cross‐sectional SEM image of PSCs revealed that the UiO‐66‐(SH)_2_‐assisted perovskite film's thickness is ≈780 nm (Figure 5b). The devices were held at maximum power point (MPP) with the applied bias of 0.90 V and 0.94 V to track the stabilized power output, resulting in stabilized PCEs of 21.23% for the pristine device and 22.72% for the UiO‐66‐(SH)_2_‐assisted device over 200 s (Figure 5c).

Furthermore, the *J–V* curves for three different concentrations of MOF are illustrated in Figure S14 (Supporting Information) and summarized in Tables S1–S3 (Supporting Information) . The best‐performing *J–V* curves of the PSCs under AM 1.5 G illumination (100 mW cm^−2^) are presented in Figure 5d, and the related photovoltaic parameters are summarized in Table S4 (Supporting Information). The pristine device shows a PCE of 22.49% with an open‐circuit voltage (*V*_oc_) of 1.158 V, a short‐circuit current density (*J*_sc_) of 24.91 mA cm^−2^, and a FF of 77.97%. In contrast, the UiO‐66‐(SH)_2_‐assisted device exhibited a higher PCE of 24.09% with improved *V*_oc_ of 1.184 V, a *J*_sc_ of 25.21 mA cm^−2^, and a FF of 80.71%. Additionally, we provide the recently reported MOF‐assisted PSCs with details listed in Table S5 (Supporting Information). The higher built‐in potentials via UiO‐66‐(SH)_2_‐assisted were beneficial for extracting the photogenerated carriers and effectively suppressing the recombination, leading to a higher *V*_oc_ (Figure S15, Supporting Information).^[^ ^38^ ^]^ The authenticity of *J*_sc_ was verified by external quantum efficiency (EQE) measurements (Figure 5e). The integrated *J*_sc_ for pristine, UiO‐66‐(SH)_2_‐assisted, UiO‐66‐MSA‐assisted, and UiO‐66‐DMSA‐assisted from the EQE curves corresponded to the measured *J_sc_* value (within a 5% error). The photovoltaic parameters for UiO‐66‐(SH)_2_‐assisted PSCs are summarized and compared with those obtained for other devices in Figure 5f and Figure S16 (Supporting Information). Optimized  *V*_oc_, FF, and PCE values were obtained for over 20 UiO‐66‐(SH)_2_‐assisted devices, with an average PCE of 23.77%, which is significantly greater than that of the pristine (22.01%), UiO‐66‐MSA‐assisted (23.07%) and UiO‐66‐DMSA‐assisted (23.15%). Additionally, the enduring stability of PSC technology is of paramount importance in various aging conditions. The unencapsulated UiO‐66‐(SH)_2_‐assisted device maintains 92.26% of its initial PCE after ≈1440 h compared with pristine devices, only maintaining 67.42% of their initial value in a dark and dry environment (20  ± 2 °C, 10% RH). With the fluorinated poly(triarylamine)(1F‐PTAA) as a hole‐transport layer based on our previous study,^[^ ^39^ ^]^ the unencapsulated UiO‐66‐(SH)_2_‐assisted device also exhibits decent thermal and humidity stability. It retains 88.5% of initial PCE after 200 h at 80 °C in the glovebox (Figure S17, Supporting Information) and the devices with 1F‐PTAA based pristine and UiO‐66‐(SH)_2_ retained ≈72% and ≈58% of the initial PCE over 300 h, while the pristine with Spiro‐OMeTAD kept only ≈24% of the initial PCE after 120 h at 85 °C and 85% RH (Figure S18, Supporting Information). Most significantly, we conducted a long‐term operational stability test of devices near the MPP under continuous light illumination with 1‐Sun intensity at 20.8 °C in an N_2_ environment. The pristine cells show a swift decline in PCE (below 42.55% of the original PCE), while the UiO‐66‐(SH)_2_‐assisted cell exhibits a prolonged lifespan, retaining over 86.44% of its initial PCE after 300 h.

## 3 Conclusion

In conclusion, our comprehensive investigation demonstrated the significant impact of thiol‐functionalized UiO‐66‐type nanoscale Zr‐based MOFs (UiO‐66‐(SH)_2_, UiO‐66‐MSA, and UiO‐66‐DMSA) on perovskite photovoltaics. The incorporation of UiO‐66‐(SH)_2_ effectively suppressed perovskite surface defects by forming the Pb‐S and the I‐H bonds, as evidenced by XPS and DFT calculations. This resulted in remarkable protection of perovskite films against degradation and substantial improvement in optoelectronic properties compared to UiO‐66‐MSA and UiO‐66‐DMSA assisted samples. Furthermore, the in situ GIWAXS measurements revealed the complete conversion of PbI_2_ to the perovskite phase and the modulation of perovskite crystallization pathways facilitated by UiO‐66‐(SH)_2_ additives. The UiO‐66‐(SH)_2_‐assisted device achieved a remarkable PCE of 24.09% and exhibited enhanced long‐term stability under ambient conditions and continuous light illumination. Overall, our findings provide valuable insights into the mechanism of MOF‐assisted perovskite film formation and the relative phase transformation process, guiding the design of desired MOF/perovskite structure for improved performance in photovoltaic applications.

## Conflict of Interest

The authors declare no conflict of interest.

## Supporting information

Supporting Information

## Data Availability

Research data are not shared.
