# Supplementary material for: Thiol‐Functionalized Conjugated Metal–Organic Frameworks for Stable and Efficient Perovskite Photovoltaics
Source: Adv Sci (Weinh). 2023 Nov 9;11(4):2305572. doi: 10.1002/advs.202305572 (PMC10811498; doi:10.1002/advs.202305572)
Supplement: Supplementary file 1 — Supporting Information [file ADVS-11-2305572-s001.pdf]

ADVANCED SCIENCE
Open Access

Supporting Information

for *Adv. Sci.*, DOI 10.1002/advs.202305572

Thiol-Functionalized Conjugated Metal–Organic Frameworks for Stable and Efficient Perovskite Photovoltaics

Xiao Liang, Mriganka Singh, Fei Wang, Patrick W. K Fong, Zhiwei Ren, Xianfang Zhou, Xuejuan Wan, Carolin M. Sutter-Fella, Yumeng Shi, Haoran Lin, Quanyao Zhu*, Gang Li* and Hanlin Hu**

Copyright WILEY-VCH Verlag GmbH & Co. KGaA, 69469 Weinheim, Germany, 2020.
DOI: 10.1002/ advs.202305572
Article type: Communication

Supporting Information

Thiol-functionalized Conjugated Metal-Organic Frameworks for Stable and Efficient Perovskite Photovoltaics

Xiao Liang[1,2], Mriganka Singh[3], Fei Wang[1,2], Patrick W. K Fong[4,5], Zhiwei Ren[4,5], Xianfang Zhou[1,2], Xuejuan Wan[6*], Carolin M. Sutter-Fella[3], Yumeng Shi[7], Haoran Lin[2], Quanyao Zhu[1*],Gang Li[4,5*], Hanlin Hu[2*]

[1]State Key Laboratory of Advanced Technology for Materials Synthesis and Processing, School of Materials Science and Engineering, Wuhan University of Technology, Wuhan, China
[2]Hoffmann Institute of Advanced Materials, Shenzhen Polytechnic, 7098 Liuxian Boulevard, Shenzhen 518055, China.
[3]Molecular Foundry Division, Lawrence Berkeley National Laboratory, Berkeley, California 94720, USA
[4]The Hong Kong Polytechnic University Shenzhen Research Institute, Guangdong, Shenzhen, 518057, China
[5]Department of Electronic and Information Engineering, Research Institute for Smart Energy (RISE), The Hong Kong Polytechnic University, Hung Hom, Kowloon, Hong Kong, China
[6]Shenzhen Key Laboratory of Polymer Science and Technology, College of Materials Science and Engineering, Shenzhen Univeristy, Shenzhen 518060, China
[7]International Collaborative Laboratory of 2D Materials for Optoelectronics Science and Technology of Ministry of Education, Institute of Microscale Optoelectronics, Shenzhen University, Shenzhen 518060, China

Experimental Section

Materials: ITO glass substrates with a sheet resistance of ca. 14 Ω sq^{-1} were purchased from OPVTECH Inc. UiO-66-$(SH)_2$, UiO-66-MSA, and UiO-66-DMSA was supplied from CHEMSOON. Formamidinium Iodide (FAI) and methylammonium bromide (MABr) were supplied from Dyesol. Methylammonium chloride (MACl), bis (trifluoromethane) sulfonimide lithium salt (Li-TFSI, 99%), 4-tert-butylpyridine (tBP, 96%), Spiro-OMeTAD (purity. 99.5%), CsI (99.99%) were supplied from Xi'an Polymer Light Technology Corp. Tin (II) chloride dehydrate ($SnCl_2 \cdot 2H_2O$), thiourea, DMF, DMSO, CB, acetonitrile were purchased from Sigma-Aldrich. PbI_2 (99.8%) was purchased from TCI. All chemicals were used as received without further treatment.

Devices Fabrications: The PSCs were fabricated on cleaned ITO substrates. ITO substrates were sequentially rinsed by sonication in detergent, deionized (DI) water, acetone, and ethanol, and finally dried in the air by nitrogen flow. Before the deposition of ETL, ITO substrates were exposed to UV–ozone for 30 min. A thin layer of SnO_2 nanoparticle film was spin-coated on the ITO substrate at 4,000 r.p.m. for 30 s to form a 50-nm-thick ETL and annealed in ambient air at 150 °C for 30 min.

Then, the perovskite layer was deposited on the SnO_2 layer by a two-step spin-coating method. The PbI_2-MOF precursor solution was prepared by dissolving 1.4 M PbI_2 into the mixed solvent of DMF and DMSO (4.5:0.5, v/v) in a nitrogen-filled glove box. The dissolved solution was filtered with the filter (0.22μm, Oriental Chemicals). The filtered PbI_2 solution added UiO-66-$(SH)_2$, UiO-66-MSA, and UiO-66-DMSA powder and sealed sonicated for 30 min. The PbI_2 precursor solution was first spin-coated on the glass at 1500 rpm for 30 s. The substrate with the newly deposited PbI_2 layer was annealed at 70 °C for 1 min. After the PbI_2 film cooled down to room temperature, 40 μL of the organic mixture solution of FAI: MACl: MABr (60: 6: 6 mg in 1 mL IPA) was spin-coated onto the PbI_2 during spinning at 1800 rpm for 30 s. When

the resulting film turned from orange to dark brown in drying, they were thermally annealed at 130 °C for 30 min under ambient conditions. Filtered spiro-OMeTAD solution (72.3 mg dissolved in 1 mL chlorobenzene) with 30 μL of tBP and 35 μL of Li-TFSI (260 mg mL^{-1}in acetonitrile) was spin-coated on the top of the perovskite layer at 4000 rpm for 30 s in a glove box after the substrates cooling down to room temperature.

Finally, 90–100 nm of gold was deposited by thermal evaporation on top of the HTL layer to complete the device, using a shadow mask to pattern the electrodes. The active area of the cells was 0.09 cm^2, which was defined by the overlapped area of the Au electrode and the ITO stripe.

Computational Details: All the calculations are performed in the framework of the density functional theory with the projector augmented plane-wave method, as implemented in the Vienna ab initio simulation package[1]. The generalized gradient approximation proposed by Perdew, Burke, and Ernzerhof is selected for the exchange-correlation potential[2]. Weak van der Waals interaction is considered by the DFT-D3 functional[3]. The cut-off energy for the plane wave is set to 400 eV. The energy criterion is set to 10−5 eV in the iterative solution of the Kohn-Sham equation. The Brillouin zone integration is performed at the Gamma point. All the structures are relaxed until the residual forces on the atoms have declined to less than 0.05 eV/Å.

GIWAXS measurement: GIWAXS measurements were performed at the Synchrotron & Printable Electronic Lab, Hoffmann Institute of Advanced Materials, Shenzhen Polytechnic with SAXSFocus 3.0 equipped with a Cu X-ray Source (8.05 keV, 1.54 Å) and a EIGER 2R 500K detector. The incidence angle is 0.5°.

In-situ GIWAXS measurement: in-situ grazing-incidence wide-angle scattering measurements (GIWAXS) was performed during thermal annealing in a custom-made spin coater. The spin coater is attached to the endstation of beamline 12.3.2 at the Advanced Light Source (ALS), Lawrence Berkeley National Laboratory. GIWAXS data were recorded every second on a 2D Pilatus 1 M detector (Dectris Ltd.). The temperature of the heating puck was recorded by a pre-

calibrated Raytek MI3 pyrometer, which controlled the annealing temperature and protocol through a pre-programmed PID loop[4]. The distance from the sample to the detector set to 270 mm. An incident photon energy of 10 keV was applied, with a corresponding wavelength of 0.124 nm. An incident angle αi of 0.5° (near the critical angle) was applied in the GIWAXS experiment, thus providing a global and strong (averaged) scattering signal for the sample. The GIWAXS data are collected during the thermal annealing process with an exposure time of 5 s.

Characterization: The *J–V* characteristics of the devices were measured using a B1500 A semiconductor parameter analyzer under the calibrated ABET Technologies SUN 2000 solar simulator equipped with an AM 1.5 filter at 100 mW cm^{-2}.

The corresponding IPCE spectrum was measured in air by a QE-R3011 system from Enli Technology Co. Ltd. (Enli).

The morphologies of PSCs were investigated by a high-resolution field emission SEM (JEOL JSM-6335F).

PL spectrum and TRPL signals of perovskite film were recorded by using Edinburgh FLSP920 spectrophotometer equipped with the excitation source of 465 nm picosecond pulsed diode laser.

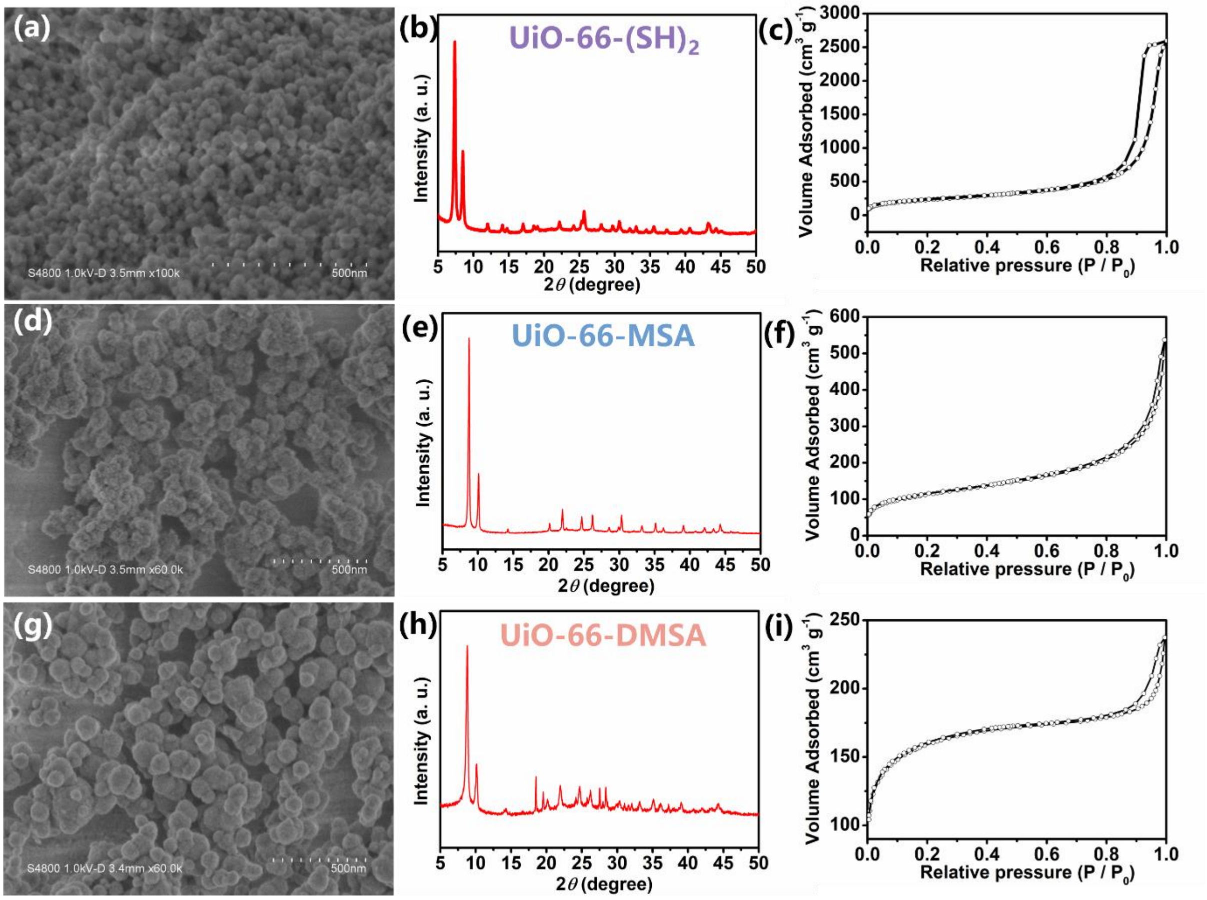

Figure S1. Scanning electron microscope (SEM) images, X-ray diffraction spectra (XRD), and (b) N_2 adsorption-desorption isotherms of (a-c) UiO-66-$(SH)_2$, (b-f) UiO-66-MSA, and (g-i) UiO-66-DMSA.

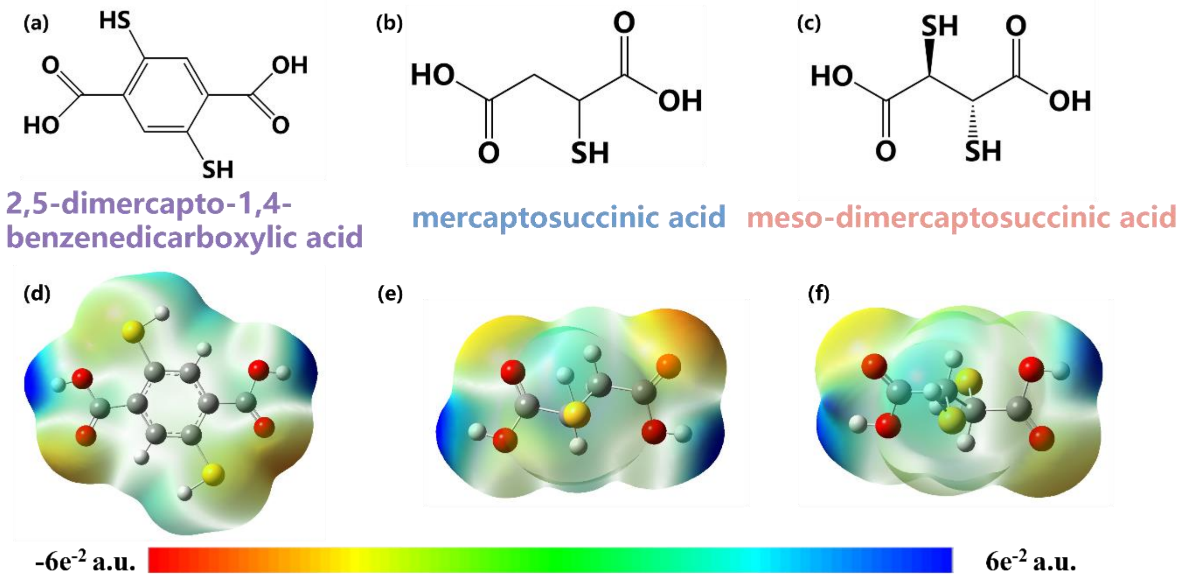

Figure S2. Small molecule organic ligand and electrostatic surface potential (ESP) for (a,d) UiO-66-$(SH)_2$, (b,e) UiO-66-MSA, and (c,f) UiO-66-DMSA.

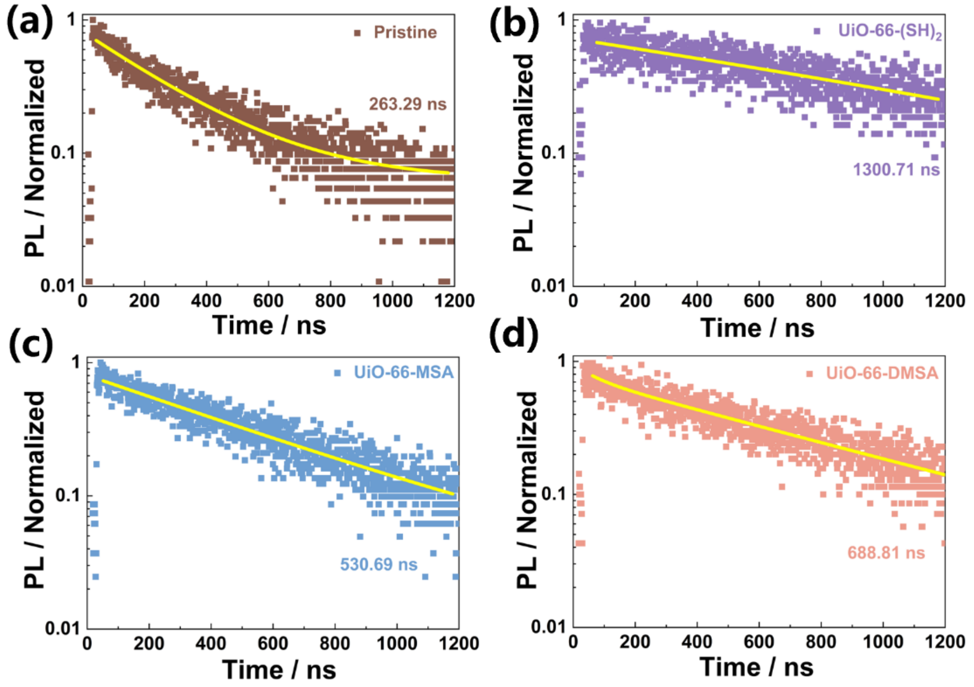

Figure S3. TRPL spectra of (a) pristine, (b) UiO-66-$(SH)_2$, (c) UiO-66-MSA, and (d) UiO-66-DMSA assisted perovskite films. Yellow solid lines are the fitted profiles using a biexponential decay function.

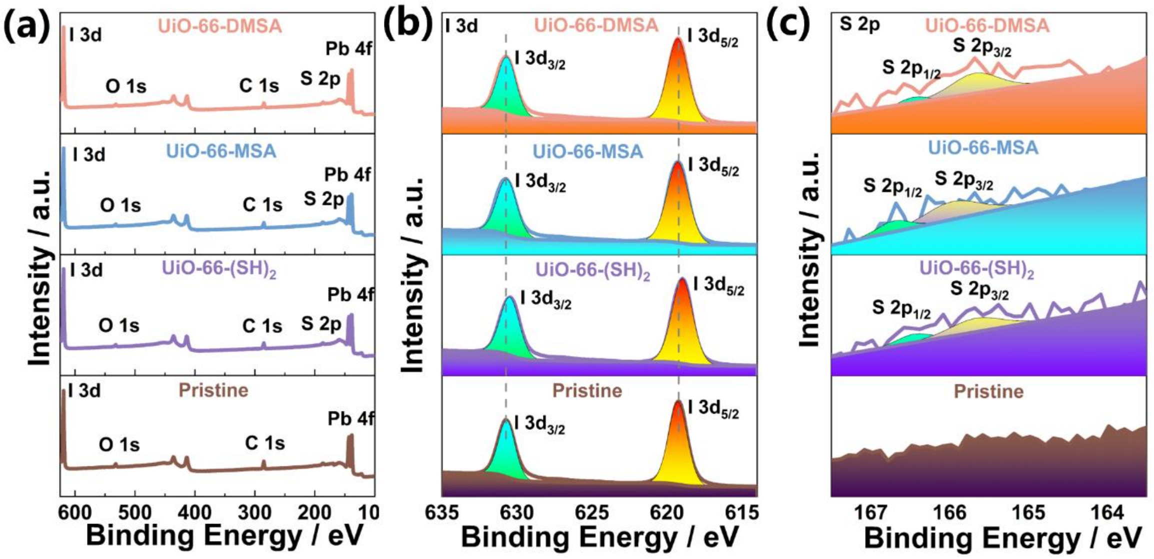

Figure S4. XPS measurements of (a) entire binding energy survey, (b) I 3d, and (c)S 2p for pristine, UiO-66-$(SH)_2$, UiO-66-MSA, and UiO-66-DMSA assisted PbI_2 film.

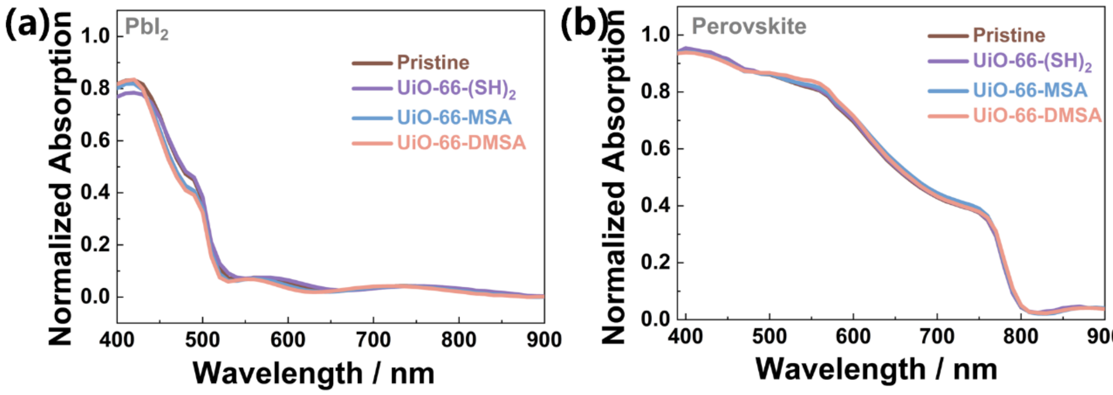

Figure S5. UV-vis of the pristine, UiO-66-$(SH)_2$, UiO-66-MSA, and UiO-66-DMSA assisted (a) PbI_2 and (b) perovskite films.

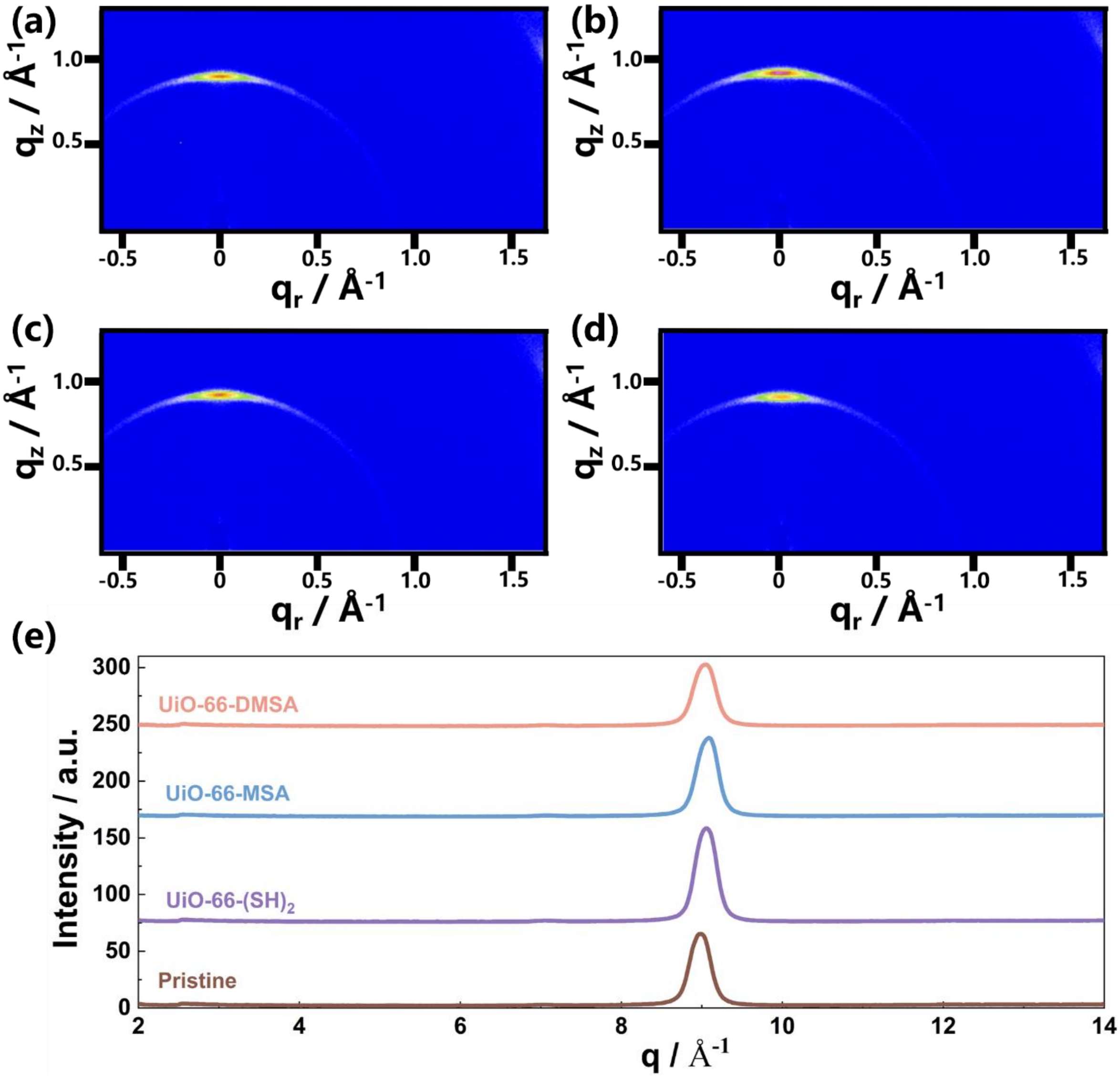

Figure S6. (a) GIWAXS images of (a) pristine and PbI_2 film formals *via* (b) UiO-66-$(SH)_2$, (c) UiO-66-MSA, and (d) UiO-66-DMSA. (e) Radial integration of PbI_2 from corresponding GIWAXS patterns.

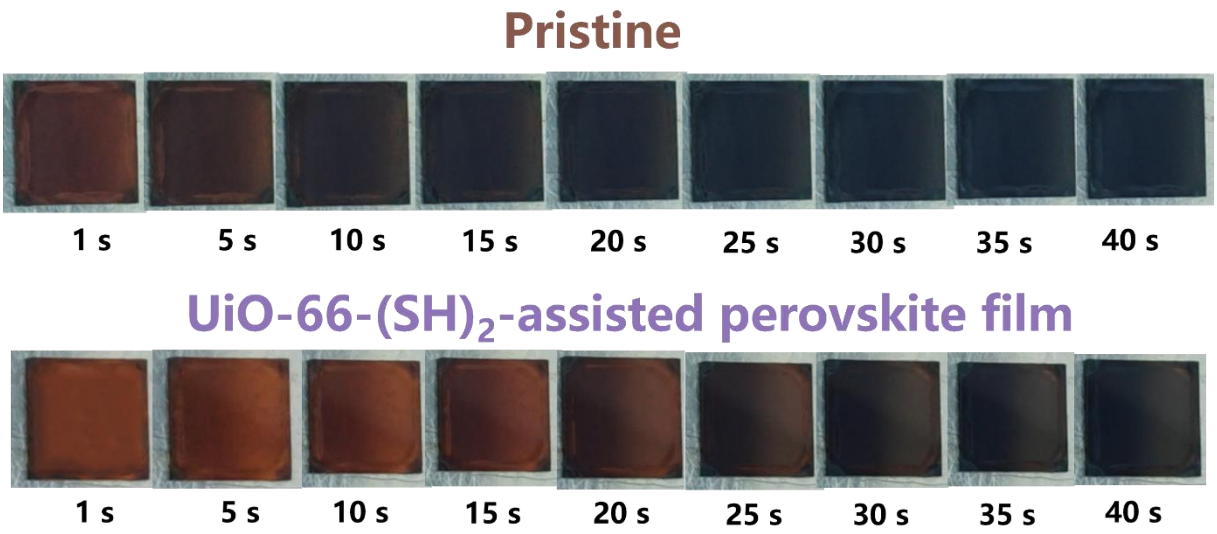

Figure S7. Photographs of perovskite films were collected at different annealing times for pristine and UiO-66-$(SH)_2$-assisted perovskite films.

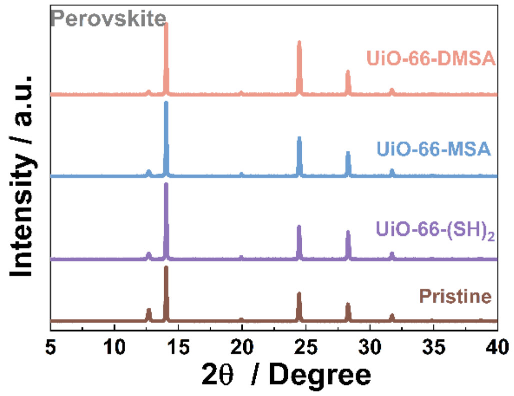

Figure S8. XRD patterns of the pristine, UiO-66-$(SH)_2$, UiO-66-MSA, and UiO-66-DMSA assisted perovskite film.

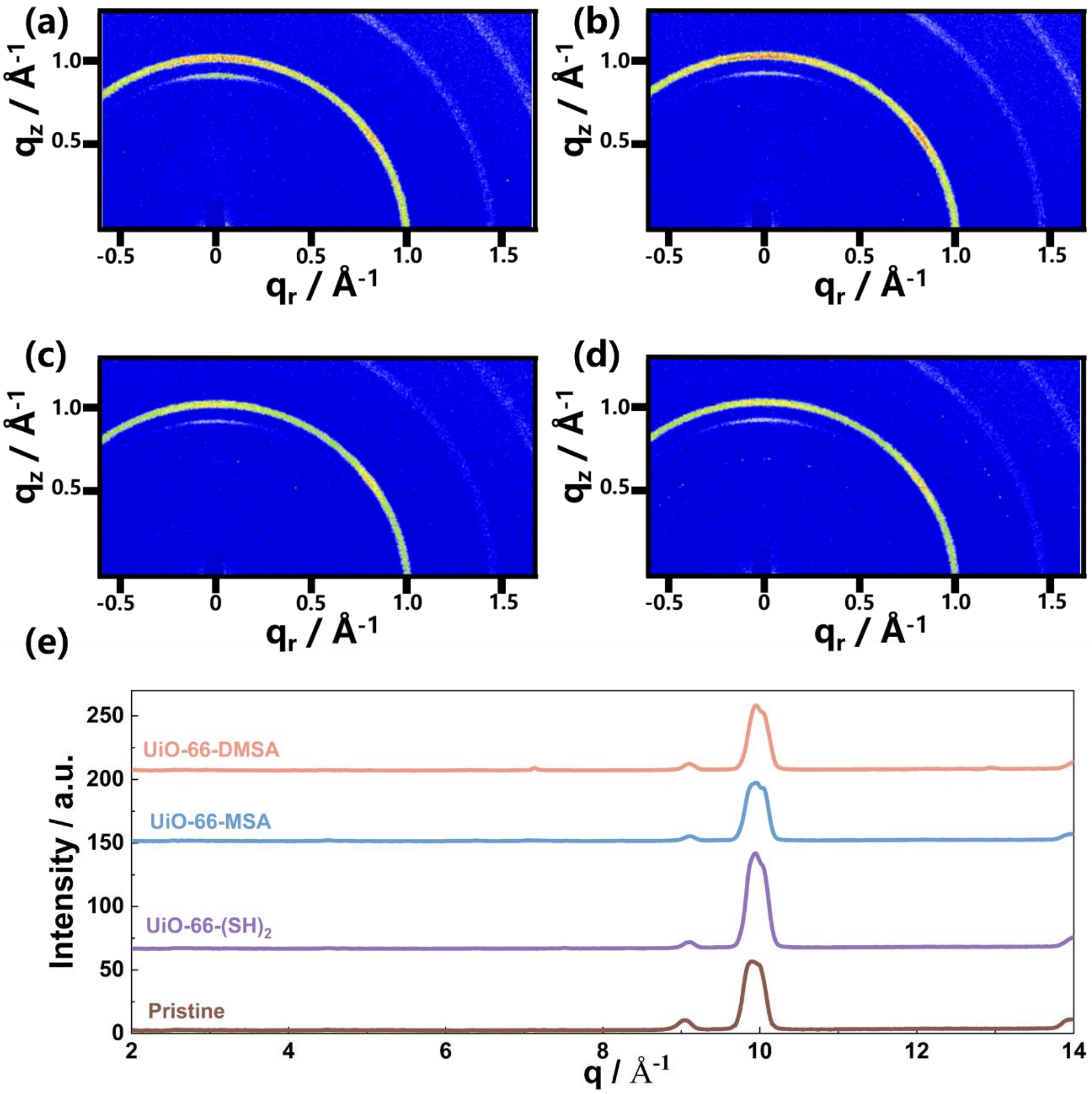

Figure S9. (a) GIWAXS images of the perovskite film (a) pristine and via (b) UiO-66-$(SH)_2$, (c) UiO-66-MSA, and (d) UiO-66-DMSA. (e) Radial integration of perovskite from corresponding GIWAXS patterns.

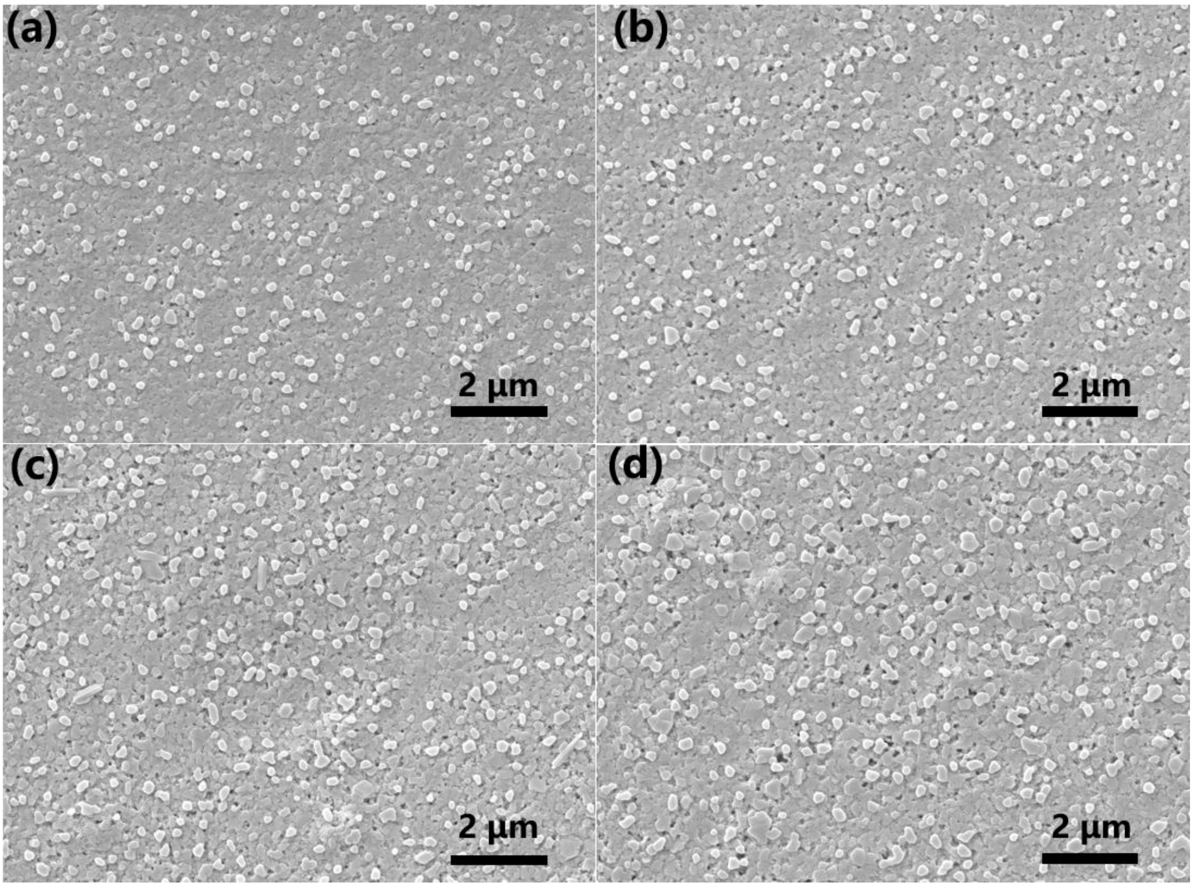

Figure S10. The plan-view Scanning electron microscope (SEM) of the (a) pristine, (b) UiO-66-$(SH)_2$, (c) UiO-66-MSA, and (d) UiO-66-DMSA assisted PbI_2 thin films.

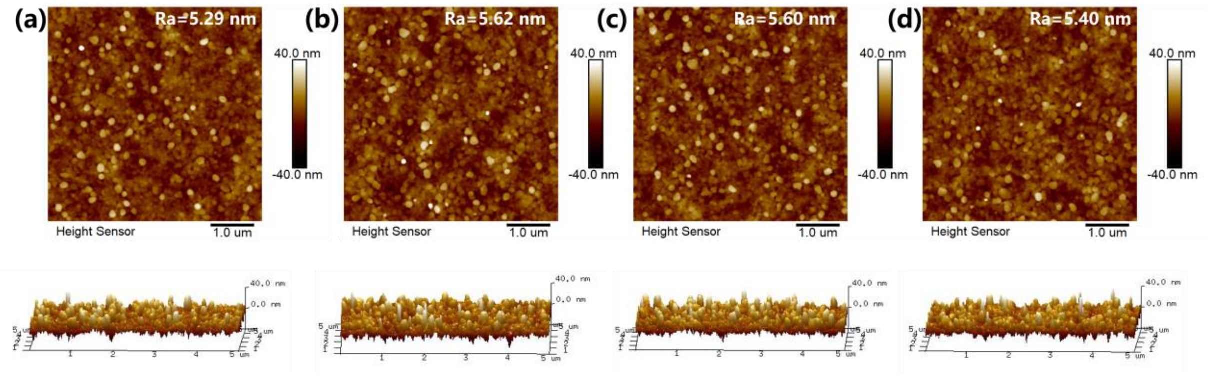

Figure S11. Atomic force microscopy (AFM) images for (a) pristine, (b) UiO-66-$(SH)_2$, (c) UiO-66-MSA, and (d) UiO-66-DMSA assisted PbI_2 films.

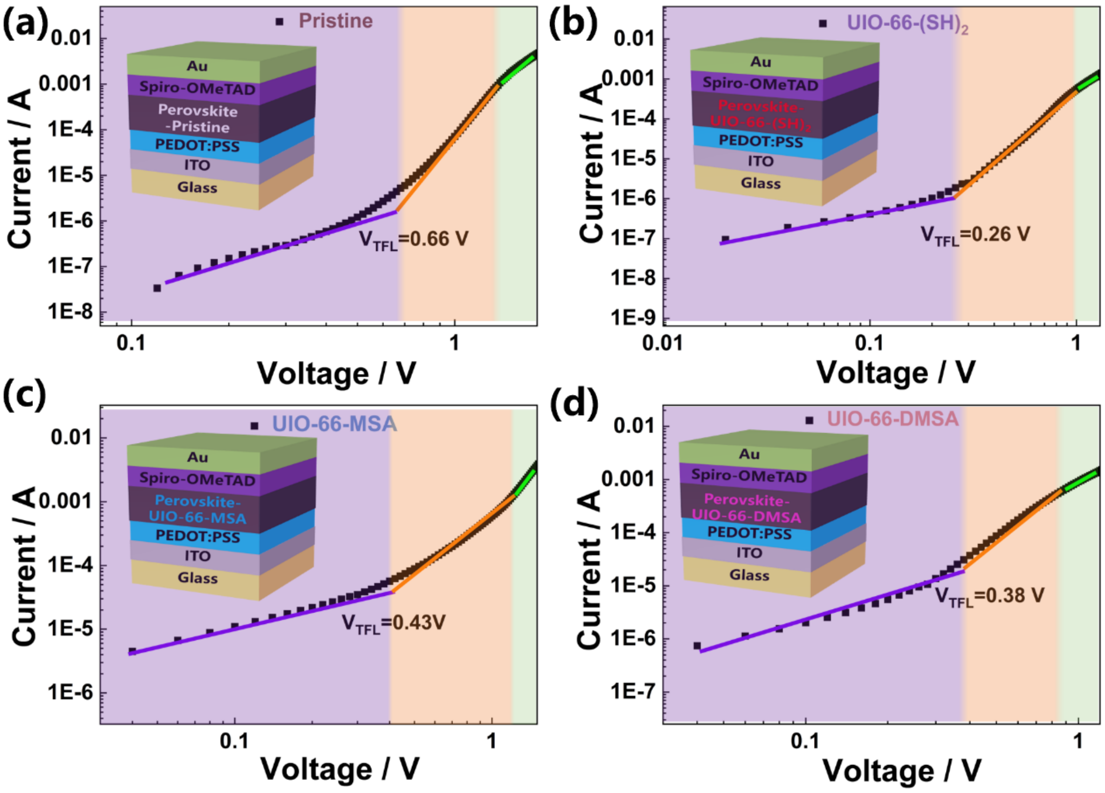

Figure S12. Space-Charge Limited Current indicates the trap-filling limited voltage (V_{TFL}) of the hole-only devices for (a) pristine, (b) UiO-66-$(SH)_2$ assisted, (b) UiO-66-MSA assisted, and (b) UiO-66-DMSA assisted.

The N_t of perovskite films was calculated using the SCLC method according to the following equation (1).

$$N_t = \frac{2\varepsilon\varepsilon_0 V_{TFL}}{eL^2} \quad (1)$$

Where e denotes elementary charge, L represents the thickness of the perovskite film, ε means the relative dielectric constant of perovskite, and ε_0 indicates the vacuum permittivity. V_{TFL} is the onset voltage of the trap-filled limit region.

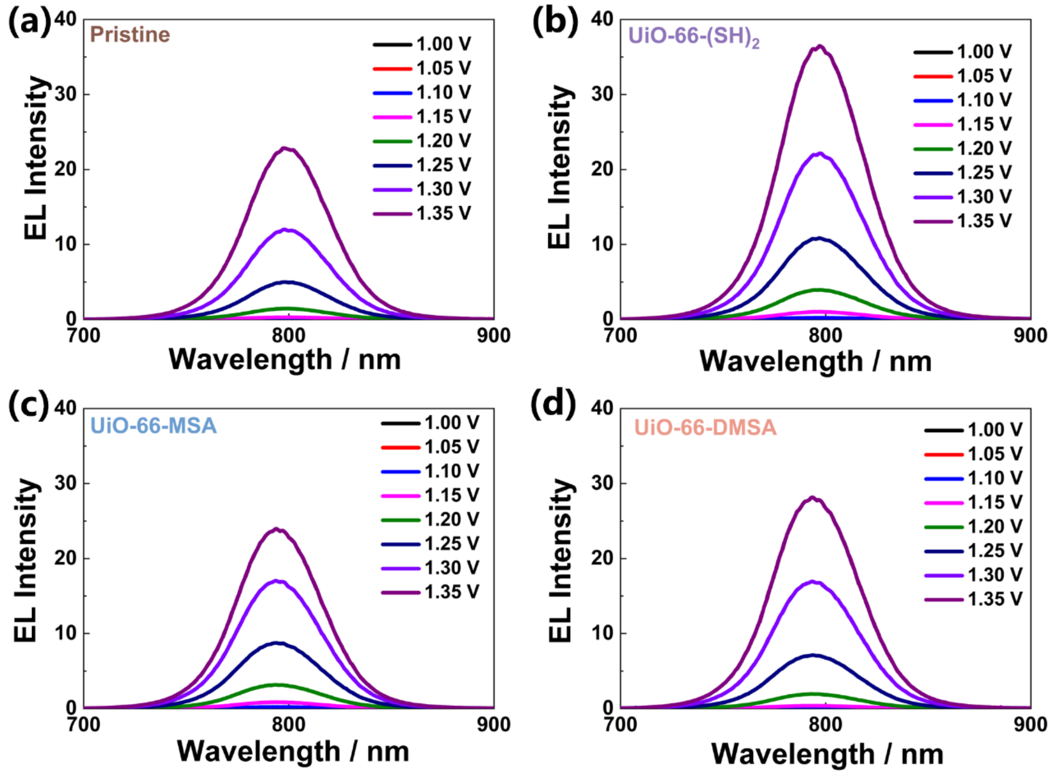

Figure S13. EL transients at different bias voltages were observed for (a) pristine, (b) UiO-66-$(SH)_2$, (c) UiO-66-MSA, and (d) UiO-66-DMSA.

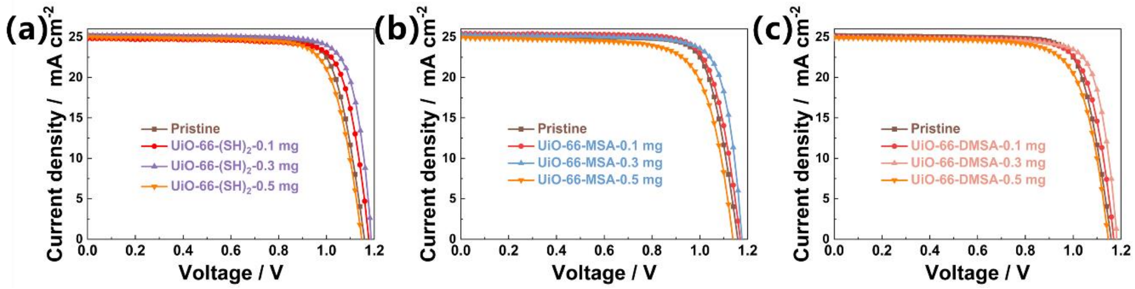

Figure S14. Current density–voltage (*J-V*) curves of the pristine and different concentrations of UiO-66-$(SH)_2$, UiO-66-MSA, and UiO-66-DMSA-assisted devices.

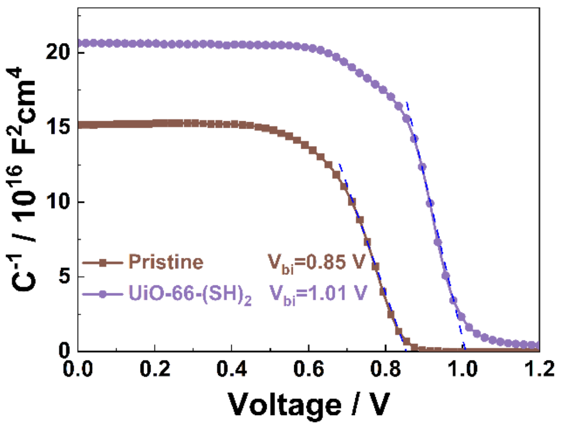

Figure S15. Mott–Schottky plots of the devices reveal the interfacial charge density for the pristine and UiO-66-$(SH)_2$-assisted devices.

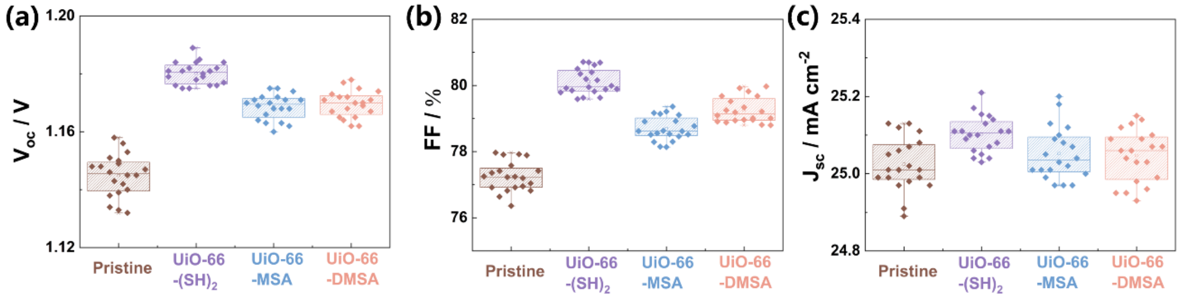

Figure S16. Statistical deviation of the photovoltaic parameters obtained from the PSCs with and without the MOF assisted (20 solar cells of each type).

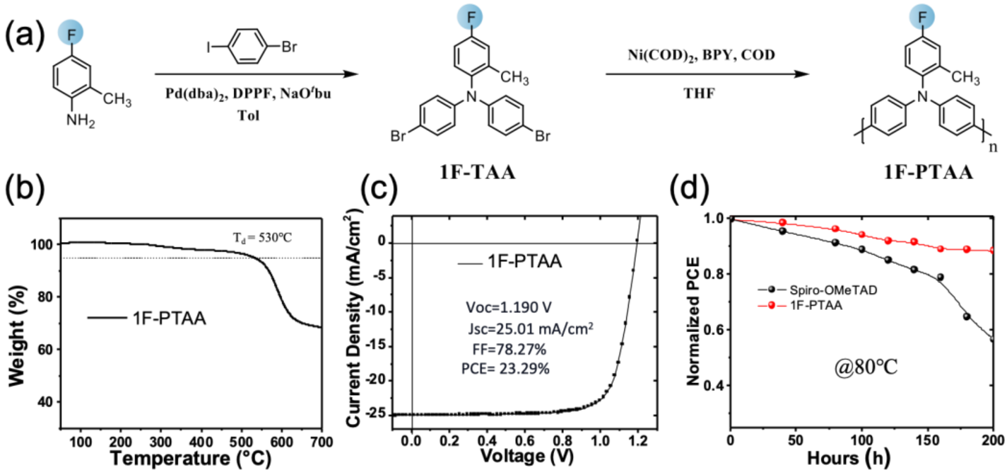

Figure S17. (a)Synthesize path for fluorinated poly(triarylamine)(1F-PTAA). (b)TGA curve of 1F-PTAA and (c) *J-V* curve for 1F-PTAA based UiO-66-$(SH)_2$-assisted device. (d) Thermal stability results for both doped Spiro-OMeTAD and 1F-PTAA based UiO-66-$(SH)_2$-assisted device at 80 °C in glove-box. 1F-PTAA based UiO-66-$(SH)_2$-assisted device still exhibts 20.61% after 200 h at 80 °C.

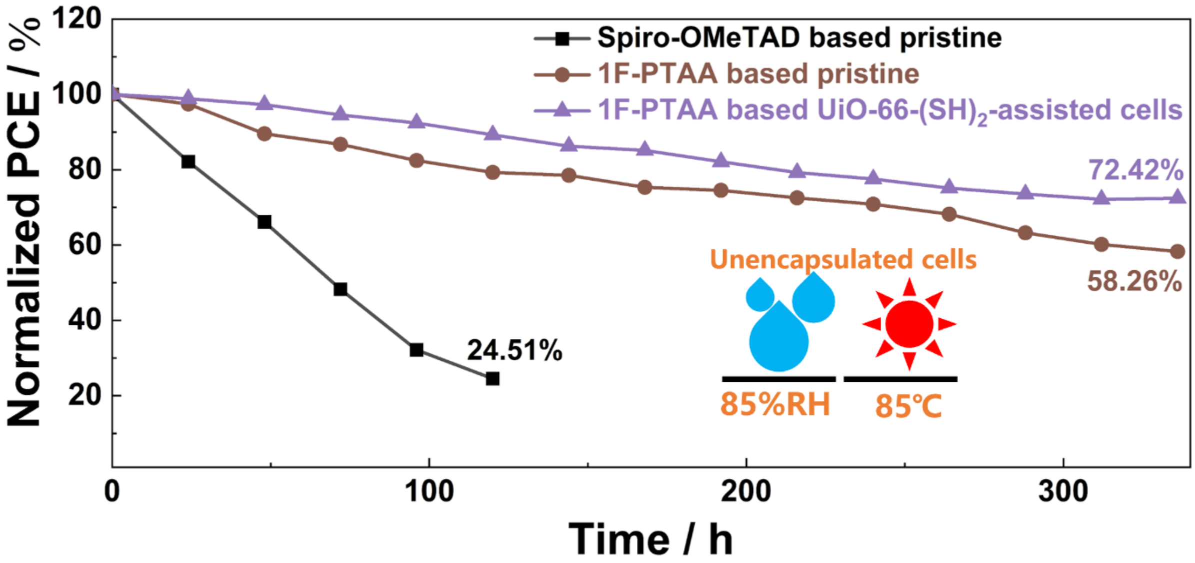

Figure S18. Thermal and humidity stability tests of unencapsulated devices both doped Spiro-OMeTAD and 1F-PTAA-based pristine, 1F-PTAA-based UiO-66-$(SH)_2$-assisted devices at 85 °C and 85% RH.

Table S1. Photovoltaic parameters of PSCs with UiO-66-$(SH)_2$-assisted under AM 1.5G illumination at 100 mW cm^{-2}.

	J_{sc} (mA cm^{-2})	V_{oc} (V)	FF (%)	PCE (%)
Pristine	24.91	1.158	77.97	22.49
UiO-66-$(SH)_2$-0.1 mg	24.81	1.178	79.08	23.11
UiO-66-$(SH)_2$-0.3 mg	25.21	1.184	80.71	24.09
UiO-66-$(SH)_2$-0.5 mg	25.01	1.153	76.06	21.93

Table S2. Photovoltaic parameters of PSCs with UiO-66-MSA-assisted under AM 1.5G illumination at 100 mW cm^{-2}.

	J_{sc} (mA cm^{-2})	V_{oc} (V)	FF (%)	PCE (%)
Pristine	24.91	1.158	77.97	22.49
UiO-66-MSA-0.1 mg	25.19	1.167	77.84	22.88
UiO-66-MSA-0.3 mg	25.06	1.172	79.36	23.30
UiO-66-MSA-0.5 mg	24.89	1.142	73.35	20.84

Table S3. Photovoltaic parameters of PSCs with UiO-66-DMSA-assisted under AM 1.5G illumination at 100 mW cm^{-2}.

	J_{sc} (mA cm^{-2})	V_{oc} (V)	FF (%)	PCE (%)
Pristine	24.91	1.158	77.97	22.49
UiO-66-DMSA-0.1 mg	25.02	1.170	77.67	22.73
UiO-66-DMSA-0.3 mg	24.93	1.178	79.94	23.47
UiO-66-DMSA-0.5 mg	24.89	1.149	74.48	21.30

Table S4. Photovoltaic parameters of PSCs with pristine and MOF-assisted under AM 1.5G illumination at 100 mW cm^{-2}. The champion PCE values are given.

	Jsc (mA cm^{-2})	Voc (V)	FF (%)	PCE (%)
Pristine	24.91	1.158	77.97	22.49
UiO-66-$(SH)_2$	25.21	1.184	80.71	24.09
UiO-66-MSA	25.06	1.172	79.36	23.30
UiO-66-DMSA	24.93	1.178	79.94	23.47

Table S5. The summarized recently reported MOFs assisted perovskite solar cells.

MOF materials	Perovskite	method	Device structures	PCE	Year	Ref.
ZrL3	CsFAMAPbI	One-step	ITO / PTAA/perovskite/$PC_{61}BM$/ ZrL3/ Ag	22.02	2020	[5]
Eu-MOF	FAMACsPbI	Two-step	ITO/SnO_2/Eu-MOF/Perovskite/ Spiro-OMeTAD/Au	22.16	2021	[6]
Cr-MOF	$CsPbI_2Br$	One-step	FTO/NiOx/Cr-MOF-$CsPbI_2Br$/ZnO@C60/Ag	17.02	2021	[7]
Zn-TTB	FAMACsPbI	One-step	FTO/TiO_2/Zn-TTB-assisted perovskite/Spiro-OMeTAD/Au	23.14	2022	[8]
ZnL	$CH3NH_3PbI_3$	One-step	FTO/TiO_2/perovskite/ZnL-perovskite/HTM/Ag	21.15	2022	[9]
PCN-224	CsFAMAPbI	One-step	FTO/TiO_2/PCN-224-perovksite / Spiro-OMeTAD/Au	22.51	2022	[10]
$Cu_3(HHTT)_2$	FASnCsIBr	One-step	ITO/NiOx/ $Cu_3(HHTT)_2$-perovskite/PCBM/BCP/Ag	22.01	2022	[11]
MOF-545	CsFAMAPbIBr	One-step	FTO/C-TiO_2/$PC_{61}BM$/ perovskite/HTM/Au	21.5	2022	[12]
Cd-Httb-BDC	CsFAMAPbBr	One-step	FTO/TiO_2/Cd-Httb-BDC-perovskite/Spiro-OMeTAD/Au,	22.18	2023	[13]
POMOF	FAMACsPbI	One-step	FTO/ETL/Perovskite/oFPEAI Spiro-OMeTAD /Au	23.3	2023	[14]
ZIF-8	FAMAI	Two-step	ITO/SnO_2/ZIF-8@FAI/ perovskite/PEAI/spiro-OMeTAD/Ag	24.08	2023	[15]
UiO-66-$(SH)_2$	FAMACsPbI	Two-step	ITO/ SnO_2/ UiO-66-$(SH)_2$-assisted perovskite/ PEAI/ Spiro-OMeTAD/ Au	24.09	2023	This work

Reference

[1] G. Kresse, D. Joubert, *Phys. Rev. B* **1999**, *59*, 1758.
[2] J. P. Perdew, K. Burke, M. Ernzerhof, *Phys. Rev. Lett.* **1996**, *77*, 3865.
[3] S. Grimme, J. Antony, S. Ehrlich, H. Krieg, *J. Chem. Phys.* **2010**, *132*, 154104.
[4] S. Pratap, F. Babbe, N. S. Barchi, Z. Yuan, T. Luong, Z. Haber, T.-B. Song, J. L. Slack, C. V. Stan, N. Tamura, C. M. Sutter-Fella, P. Müller-Buschbaum, *Nat. Commun.* **2021**, *12*, 5624.
[5] S. Wu, Z. Li, M.-Q. Li, Y. Diao, F. Lin, T. Liu, J. Zhang, P. Tieu, W. Gao, F. Qi, X. Pan, Z. Xu, Z. Zhu, A. K.-Y. Jen, Nat. Nanotechnol. 2020, 15, 934.
[6] J. Dou, C. Zhu, H. Wang, Y. Han, S. Ma, X. Niu, N. Li, C. Shi, Z. Qiu, H. Zhou, Y. Bai, Q. Chen, Adv. Mater. 2021, 33, DOI 10.1002/adma.202102947.
[7] S. Yuan, Y. Xian, Y. Long, A. Cabot, W. Li, J. Fan, Adv. Funct. Mater. 2021, 31, DOI 10.1002/adfm.202106233.
[8] J. Wang, J. Zhang, S. Gai, W. Wang, Y. Dong, B. Hu, J. Li, K. Lin, D. Xia, R. Fan, Y. Yang, Adv. Funct. Mater. 2022, 32, DOI 10.1002/adfm.202203898.

[9] C. Li, J. Qiu, M. Zhu, Z. Cheng, J. Zhang, S. Xiang, X. Zhang, Z. Zhang, Chem. Eng. J. 2022, 433, 133587.
[10] Y. Liu, T. Liu, X. Guo, M. Hou, Y. Yuan, S. Shi, H. Wang, R. Zhang, C. Galiotis, N. Wang, Adv. Funct. Mater. 2023, 33, DOI 10.1002/adfm.202210028.
[11] J. Cao, C.-K. Liu, V. Piradi, H.-L. Loi, T. Wang, H. Cheng, X. Zhu, F. Yan, ACS Energy Lett. 2022, 7, 3362.
[12] Y. Dong, J. Zhang, Y. Yang, J. Wang, B. Hu, W. Wang, W. Cao, S. Gai, D. Xia, K. Lin, R. Fan, Nano Energy 2022, 97, 107184.
[13] Y. Dong, S. Gai, J. Zhang, R. Fan, B. Hu, W. Wang, W. Cao, J. Wang, K. Zhu, D. Xia, L. Geng, Y. Yang, J. Energy Chem. 2023, 77, 1.
[14] Y. Dong, J. Zhang, W. Wang, B. Hu, D. Xia, K. Lin, L. Geng, Y. Yang, Small 2023, 19, DOI 10.1002/smll.202301824.
[15] W. Sheng, J. He, J. Yang, Q. Cai, S. Xiao, Y. Zhong, L. Tan, Y. Chen, Adv. Mater. 2023, 35, DOI 10.1002/adma.202301852.
